# Antimicrobial peptide human β-defensin-2 improves *in vitro* cellular viability and reduces pro-inflammatory effects induced by enteroinvasive *Escherichia coli* in Caco-2 cells by inhibiting invasion and virulence factors’ expression

**DOI:** 10.3389/fcimb.2022.1009415

**Published:** 2022-10-13

**Authors:** Alessandra Fusco, Vittoria Savio, Brunella Perfetto, Roberto Mattina, Giovanna Donnarumma

**Affiliations:** ^1^Department of Experimental Medicine, University of Campania “Luigi Vanvitelli”, Naples, Italy; ^2^Department of Biomedical Surgical and Dental Sciences, University of Milan, Milan, Italy

**Keywords:** intestinal epithelium, microbiota, EIEC, AMPs, HBD-2

## Abstract

*Escherichia coli* is one of the commensal species most represented in the intestinal microbiota. However, there are some strains that can acquire new virulence factors that enable them to adapt to new intestinal niches. These include enteroinvasive *E. coli* (EIEC) that is responsible for the bacillary dysentery that causes severe diarrheal symptoms in both children and adults. Due to the increasing onset of antibiotic resistance phenomena, scientific research is focused on the study of other therapeutic approaches for the treatment of bacterial infections. A promising alternative could be represented by antimicrobial peptides (AMPs), that have received widespread attention due to their broad antimicrobial spectrum and low incidence of bacterial resistance. AMPs modulate the immune defenses of the host and regulate the composition of microbiota and the renewal of the intestinal epithelium. With the aim to investigate an alternative therapeutic approach, especially in the case of antibiotic resistance, in this work we created a line of intestinal epithelial cells able to express high concentrations of AMP human β-defensin-2 (HBD-2) in order to test its ability to interfere with the pathogenicity mechanisms of EIEC. The results showed that HBD-2 is able to significantly reduce the expression of the proinflammatory cytokines by intestinal epithelial cells, the invasiveness ability of EIEC and the expression of invasion-associated genes.

## Introduction

The intestinal microbiota consists of a community of about 100 trillion mutualistic microorganisms that contributes to development of the intestine and the homeostasis of the organism by regulating various metabolic functions of the host (Fontaine and Kohl, 2020), such as digestion and the supply of nutrients (Fusco et al., 2017; Fontaine and Kohl, 2020). One of the most represented species in the intestinal microbiota is *Escherichia coli*, a Gram-negative bacillus that, as commensal, colonizes especially the lower tract of the human intestine (Croxen and Finlay, 2010).

Nonetheless, some strains of *E. coli*, through the horizontal transfer of plasmid DNA, islets of pathogenicity and transposons and through the action of bacteriophages, are able to acquire virulence factors which give it different characteristics compared to commensal species (Croxen and Finlay, 2010). These acquired virulence factors confer the ability to cause various disorders of the gastrointestinal and urinary tract, the respiratory system, and central nervous system in the host (Croxen and Finlay, 2010). Based on the difference in their pathogenic mechanisms, they have been classified five distinct groups: enteroaggregative *E. coli*, enterohemorrhagic *E. coli*, enteroinvasive *E. coli* (EIEC), enteropathogenic *E. coli*, and enterotoxigenic *E. coli* (Croxen and Finlay, 2010). Among these, EIEC is responsible for the so-called bacillary dysentery that causes diarrheal symptoms similar to shigellosis in both children and adults, particularly in low-income countries and poor hygiene conditions (Pasqua et al., 2017). Bacillary dysentery is characterized by bloody and purulent stool with leucocytes’ presence, accompanied by fever, abdominal cramps, and sometimes vomiting.

EIEC invades the colonic mucosa of human hosts, proliferates intracellularly, and spreads to adjacent cells, destroying the intestinal epithelial barrier and causing a strong acute inflammatory response with abscess formation and colonic ulceration. The entire repertoire of genes required for the invasion of host cells is grouped into a 230 kb virulence plasmid found in both EIEC and *Shigella* strains (Moreno et al., 2009). The antibiotic resistance is an ever-expanding phenomenon affecting public health worldwide (Fusco et al., 2021). Therefore, research is focused on the development of alternative therapies. In this context, AMPs, heterogeneous and amphipathic peptides synthesized as propeptides and cleaved by specific proteases in their active form, have received widespread attention due to several advantages observed compared with traditional antibiotics, as: broad antimicrobial spectrum, low frequency to drug resistance, absence of toxic residues formation (Gong et al., 2021), ease of synthesis due to their short size (Hansen et al., 2020), rapid killing activity and the ability to act on bacteria without being affected by resistance mechanisms. Furthermore, AMPs modulate the immune defenses of the host and regulate the composition of microbiota (which conversely is often destroyed by conventional antibiotic therapies) and the renewal of the intestinal epithelium (Gubatan et al., 2021).

It has been shown that the interactions between AMPs and microbiota are bidirectional; the appropriate production of AMPs is indicative of a healthy microbiota, and conversely, the metabolites of microbial origin regulate the function of the host cell and the production of AMPs (Ostaff et al., 2013). Recently, we created a line of intestinal epithelial cells (Caco-2) able to stably express high concentrations of AMP human β-defensin-2 (HBD-2) (Fusco et al., 2017; Fusco et al., 2021; Fusco et al., 2021). HBD-2 is an inducible and most abundant AMP with a molecular mass of 4–6 kD, identified for the first time in psoriatic lesions, but present in various other epithelia, oral cavity, gingival epithelial cells, the paranasal sinuses, cornea, and in intestinal, respiratory and urogenital epithelial cells. HBD-2 is induced by endogenous stimuli, infections, or wounds that acts as an endogenous antibiotic in the defense against Gram-negative bacteria. The mechanism by which HBD-2 acts against bacterial cell involves an interaction with specific surface components, lipopolysaccharide (LPS) of Gram-negative and theicoic acids of Gram-positive, which leads to an alteration of the cell permeability both through the formation of pores and a cleansing effect that destabilizes the bonds of the surface cell layers. Thus there is a leakage of cytoplasmic components and consequent osmotic lysis of the bacterial cell (Donnarumma et al., 2016; Fusco et al., 2017).

Aiming to offer an alternative therapeutic approach to EIEC infections, especially in the case of antibiotic resistance, this work evaluates the ability of HBD-2 to interfere with the pathogenic mechanisms of EIEC, particularly the inflammatory response induced in the host, and with its invasiveness ability, also associated with the expression of invasion-related virulence factors.

## Materials and methods

### Transfection

As already described (Croxen and Finlay, 2010), total RNA was extracted using a High Pure RNA Isolation Kit (Roche Diagnostics) from primary cultures of human keratinocytes stimulated with the LPS of *Pseudomonas aeruginosa* and TNF-α to obtain a high production of HBD-2. The primary keratinocytes were chosen for their ability, unlike some immortalized cell lines, to respond very effectively to stimulation and to produce large amounts of the AMP.

It was subsequently transcribed into complementary cDNA using random hexamer primers (Random hexamers, Roche) following the manufacturer’s instructions. A pair of degenerate primers, designed on their specific amino acid sequence (respectively HBD-2 for: 5’-CCAGCCATCAGCCATGAGGGT-3’; HBD-2 rev-5’- GGAGCCCTTTCTGAATCCGCA-3’ 254 bp), were used to amplify, by real-time PCR, gene coding HBD-2 with FastStart High Fidelity (Roche Diagnostics). The amplified DNA fragments were subjected to restriction and sequencing analysis and cloned into the pEF/V5-His TOPO (Invitrogen) vector using the T4 DNA Ligase (Invitrogen), in accordance with the manufacturer’s protocol, and then transformed into *E. coli* TOP 10 (Invitrogen). The cloning vector pEF/V5-His TOPO-HBD-2 was extracted from the bacterial culture and amplified using QIAprep Spin Midiprep Kit (Qiagen). Caco-2 cells were transfected using the IBAfect reagent (IBA), according to the manufacturer’s manual. Briefly, 3 x 10^5^ cells were seeded in six-well plates, and immediately after seeding, plasmids conjugated with the transfection reagent were added. The mixture was incubated for 24 and 48 hours. After incubation, the success of the experiment was verified by the extraction of mRNA from treated cells and the amplification of HBD-2 gene by PCR. Cell-free supernatants of the transfected cells were recovered by centrifugation and assayed for the HBD-2 concentration by an enzyme-linked immunosorbent assay (Phoenix Pharmaceuticals, Inc.). For blasticidin selection, untransfected and transfected cells were cultured at 37°C and 5% CO_2_ for 14 days in the presence of the following increasing concentrations of blasticidin S (***In vivo*** Gen): 5, 10, 20, 50, 100, and 250 μg/ml. Thereafter, 3-[4,5-dimethylthiazol-2-yl]-2,5 diphenyl tetrazolium bromide (MTT) labeling reagent (Sigma) was added at a final concentration of 0.5 mg/ml. After 4 hours, a solubilization solution was added to each well, and the plates were incubated overnight. Spectrophotometric absorbance was measured using a microplate reader (ELISA) at a wavelength of 570 nm (Fusco et al., 2017).

### Cell culture

Caco-2 cells (Human Caucasian colon adenocarcinoma cells, ATCC^®^ HTB-37™) were routinely cultured in Dulbecco’s modified Eagle medium (DMEM, Gibco) supplemented with 1% PenStrep, 1% glutamine, and 10% fetal calf serum (Gibco) at 37°C and 5% CO_2_. After transfection, as described in our previous work (Croxen and Finlay, 2010), the cells were grown in the presence of 250 μg/ml blasticidin S in a sterile 25 cm^2^ flask at a concentration of 3 x 10^5^ to confluence for 21 days to reach full differentiation and polarization. The culture medium was changed every two days.

### Bacterial strain

EIEC (ATCC^®^ 43893™) was cultured in Luria-Bertani broth (Oxoid; Unipath, Basingstoke, UK) at 37°C for 18 h. Before the experiments, the bacterial culture at exponential growth phase (~0.5 O.D.) was centrifuged 3 times for 5 minutes at 4000 x *g* to remove any residual culture supernatant and the pellet was resuspended in DMEM without antibiotics.

### Cell infection and Alamar Blue assay

Caco-2 and Caco-2/HBD-2 cells, cultured as previously described, were seeded into six-well plates and then infected with exponentially growing bacteria at a multiplicity of infection (MOI) of 100 for 6 h and 24 h at 37°C in 5% CO_2_ in DMEM without antibiotics. After 6 hours of infection, resazurin was added to the cells at a concentration of 0.5 mg/ml and incubated for 4 hours to obtain the percentage values of viable cells. Data were expressed as percentage of reduced AlamarBlue (%AB_red_), calculated correlating the absorbance values and the molar extinction coefficients of the dye under a double wavelength reading (570 nm and 600 nm), as manufacturer’s instructions. The equation applied is shown below, in which: λ = absorbance, s = sample, and c = control:


$$\%AB_{red}=100\cdot \frac{\left(117,216\lambda_{sample@570\;nm}-\;80,586\lambda_{sample@600\;nm}\right)}{\left(155,677\lambda_{control@600\;nm}-\;14,652\lambda_{control@570\;nm}\right)}$$

### Real time PCR

Cells infected with EIEC for 6 hours as previously described were washed with sterile PBS three times, and to evaluate the expression of pro- and anti-inflammatory cytokines, total RNA was extracted using a High Pure RNA Isolation Kit (Roche Diagnostics). Two hundred nanograms of total cellular RNA were reverse-transcribed (Expand Reverse Transcriptase, Roche) into complementary DNA (cDNA) in accordance with the manufacturer’s instructions. Real-time PCR for IL-6, IL-8, TNF-α, IL-1α, and IL-1β was carried out with the LightCycler FastStart DNA Master SYBR Green kit using 2 µL cDNA, corresponding to 10 ng of total RNA in a 20 μL final volume, 3mM MgCl_2_, and 10 μM sense and antisense primers (**Table 1**). After amplification, melting curve analysis was performed by heating the sample to 95°C for 15s with a temperature transition rate of 20°C/s, cooling it to 60°C for 15s with a temperature transition rate of 20°C/s, and then heating it at 0.1°C/s to 95°C. The results were then analyzed using LightCycler software (Roche Diagnostics). The standard curve of each primer pair was established with serial dilutions of cDNA. All PCR reactions were run in triplicate. The specificity of the amplification products was verified by electrophoresis on a 2% agarose gel and through visualization by ethidium bromide staining (Fusco et al., 2017).

**Table 1:** Primers sequences and amplification programs.

Gene	Primers sequence	Conditions	Product size (bp)
*IL-6*	5’-ATGAACTCCTTCTCCACAAGCGC-3’ 5’-GAAGAGCCCTCAGGCTGGACTG-3’	5’’at 95°C, 13” at 56°C, 25’’at 72°C for 40 cycles	628
*IL-8*	5’-ATGACTTCCAAGCTGGCCGTG-3’ 5’-TGAATTCTCAGCCCTCTTCAAAAACTTCTC-3’	5’’at 94°C, 6” at 55°C, 12’’at 72°C for 40 cycles	297
*IL-1β*	5’-GCATCCAGCTACGAATCTCC-3’ 5’-CCACATTCAGCACAGGACTC-3’	5’’at 95°C, 14” at 58°C, 28’’at 72°C for 40 cycles	708
*IL-1α*	5’- CATGTCAAATTTCACTGCTTCATCC-3’ 5’- GTCTCTGAATCAGAAATCCTTCTATC -3’	5”at 95°C, 8”at 55°C, 17”at 72°C for 45 cycles	420
*TNF-α*	5’- CAGAGGGAAGAGTTCCCCAG -3’ 5’- CCTTGGTCTGGTAGGAGACG -3’	5’’at 95°C, 6” at 57°C, 13’’at 72°C for 40 cycles	324
*ipaB*	5’- CCGGCAATTCCTTCATGGAAC -3’ 5’- AGTTGAGAAGAAAAATTCTTG -3’	5’’at 95°C, 6” at 50°C, 12’’at 72°C for 40 cycles	310
*ipaC*	5’- GTCACACAAGTAGGTATAACG -3’ 5’- TCTGGGTGTCAATTTTATCCT -3’	5’’at 95°C, 6” at 50°C, 12’’at 72°C for 40 cycles	301
*icsB*	5’- TGCATCAAGTCTTTCGGCTGT -3’ 5’- AACTCAATTCAACACTCTTTC -3’	5’’at 95°C, 7” at 55°C, 14’’at 72°C for 40 cycles	355
*virF*	5’- CATTTCAACACTCCTATTC -3’ 5’- AACTAAGAGAAGAAGCTATCG -3’	5’’at 95°C, 4” at 53°C, 8’’at 72°C for 40 cycles	208
*virB*	5’- CAGCAAAAGAGCATAGCATC -3’ 5’- AGAGATTCATTAGCCTTTTC -3’	5’’at 95°C, 5” at 53°C, 10’’at 72°C for 40 cycles	251
*icsA*	5’- GAGTCAATCTACCCATAATC -3’ 5’- GTGTTCCATCATCTTGTTTAC -3’	5’’at 95°C, 7” at 50°C, 14’’at 72°C for 40 cycles	342
*ial*	5’- CTGGATGGTATGGTGAGG-3’ 5’- GGAGGCCAACAATTATTTCC-3’	5’’at 95°C, 7” at 55°C, 13’’at 72°C for 40 cycles	320

### ELISA assay for pro- and anti-inflammatory cytokines

Caco-2 and Caco-2/HBD-2 cell monolayers were infected for 24 hours at 37°C with EIEC as previously described. At the end of the experiment, supernatants were harvested, and the presence of cytokines IL-6, IL-8, IL-1β, TNF-α, and TGF-β was analyzed by enzyme-linked immunosorbent assay (ELISA; ThermoFischer Scientific Inc.).

### Bacterial internalization assay

Transfected and untransfected Caco-2 cells were infected with EIEC as previously described. After 3 h of incubation at 37°C, cell supernatants were collected, and infected monolayers were extensively washed with sterile PBS and further incubated for another two hours in the DMEM medium, supplemented with gentamicin sulphate (250 μg/ml) (Sigma-Aldrich) to kill the extracellular bacteria. At the end of the experiments, infected monolayers were extensively washed in PBS and then lysed with a solution of 0.1% Triton X-100 (Sigma-Aldrich) in PBS for 10 minutes at room temperature to count internalized bacteria. The aliquots of cell lysates were serially diluted and plated on Luria-Bertani agar (OXOID) and incubated at 37°C overnight to quantify viable intracellular bacteria (CFUs/ml). The efficiency was calculated as the ratio of the number of cell-internalized bacteria with the number of bacteria used to infect the cell monolayers.

### Analysis of invasion encoding genes’ expression

Supernatants collected after 3 hours of bacterial internalization assay were used to extract total bacterial RNA with Tri-Reagent^®^, following the manufacturer’s instructions. The expression of *ipaB*, *ipaC*, *icsB*, *virF*, *virB*, *icsA*, and *ial* genes (**Table 1**) (Bando et al., 2010; Farajzadeh-Sheikh et al., 2020) was evaluated by real-time PCR as described in 2.5 section.

### Statistical analysis

Significant differences among the groups were assessed through two-way ANOVA by using GraphPad Prism 6.0, and the comparison between the means was calculated by the student’s t-test. The data were expressed as means ± standard deviation (SD) of the three independent experiments.

## Results

### HBD-2 downregulates the proinflammatory cytokines’ expression e improves cell viability

Transfected and untransfected Caco-2 cells were infected for 6 and 24 hours with EIEC to analyze the ability of HBD-2 to increase cell survival to infection by reducing the inflammatory state induced by EIEC.

**Figure 1:**
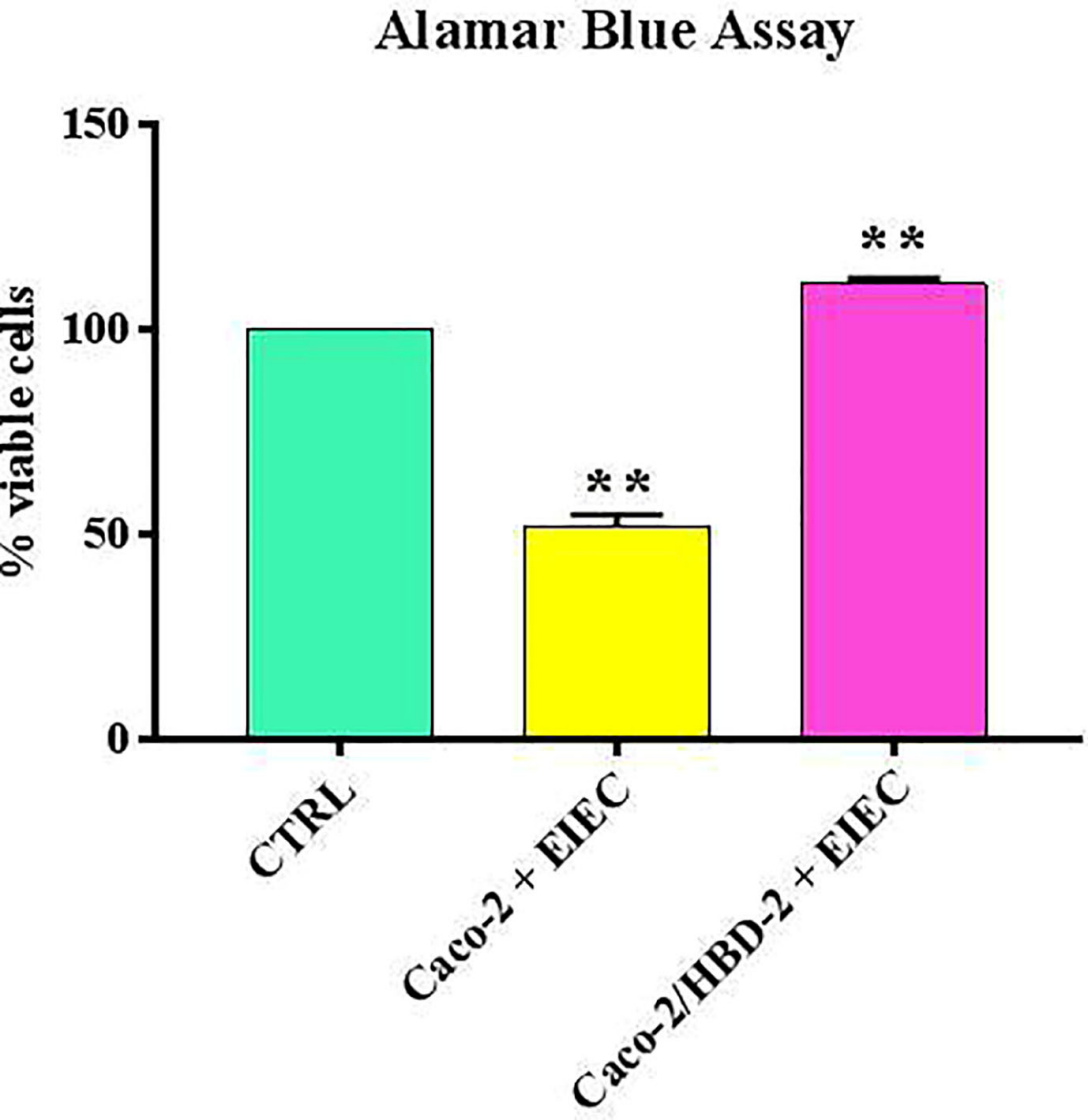
Comparison between % of viable cells in uninfected cells (ctrl), transfected and untransfected cells after 6 hours of infection with EIEC. Data are representative of three different experiments ± SD Significant differences are indicated by **p<0.01.

**Figure 2:**
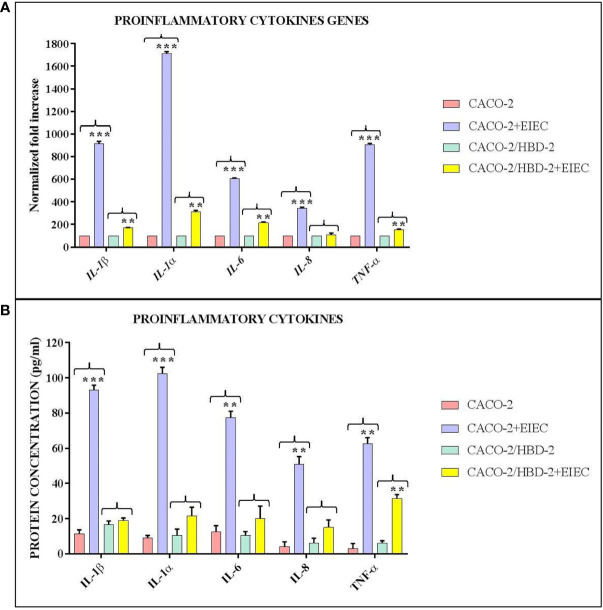
Comparison between relative gene expression **(A)** and protein concentration **(B)** in transfected and untransfected Caco-2 cells infected with EIEC. Data are representative of three different experiments ± SD and are expressed as **(A)** percentage of expression of the relative mRNAs in each group compared to uninfected cells (ctrl), arbitrarily assigned as 100%, or **(B)** as effective protein concentration. Significant differences are indicated by *p<0.05, **p<0.01, ***p<0.001.

Our results shows that after 6 hours of infection, the percentage of metabolically active cells is much higher in Caco-2/HBD-2 than in untransfected Caco-2 (**Figure 1**; **Table 2**). We can therefore attribute this greater cellular resistance to the decrease in EIEC’s ability to induce the production of proinflammatory cytokines, in fact as shown in **Figures 2A, B**, the presence of HBD-2 is able to significantly reduce the expression of the genes encoding IL-1 α, IL-1β, TNF-α, IL-6 and IL-8 (*p*<0.01) (**Figure 2A**) and the production of related proteins (*p*<0.01).

**Table 2:** % of AB_RED_ in Caco-2 and Caco-2/HBD-2 cells infected for 6 hours with EIEC.

Sample	% AB_RED_
**Caco-2**	51 ± 4
**Caco-2/HBD-2**	113 ± 6

### HBD-2 reduces the invasiveness of EIEC by downregulating invasive factors’ expression

Assuming that an increased cell survival associated with a reduced inflammatory state could also be due to a decrease in the intracellular number of bacteria, the invasive ability of EIEC in the presence/absence of HBD-2 was also evaluated by invasiveness assay and relative CFU/ml counting, and by analysis of expression of invasion-related genes.

**Figure 3:**
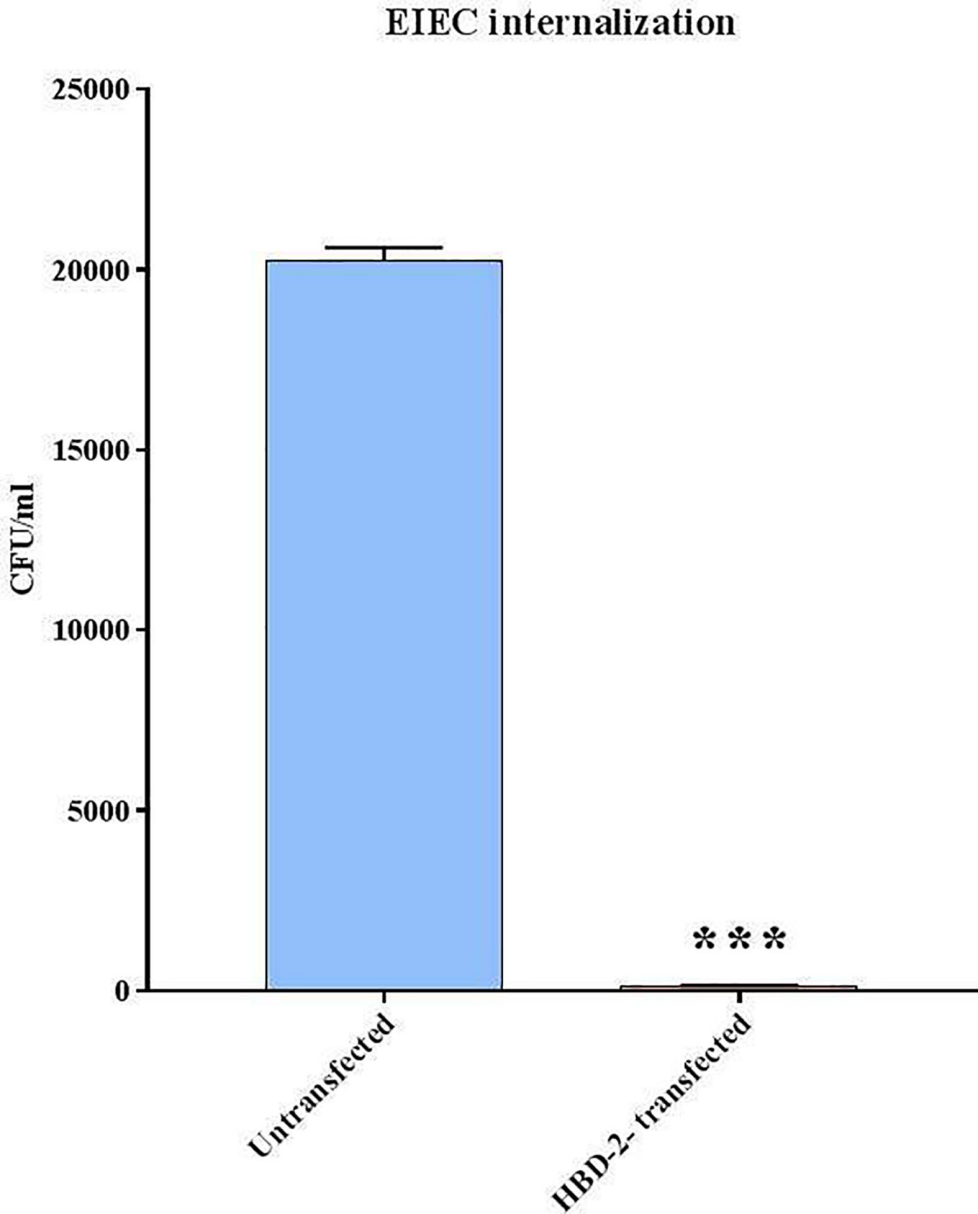
EIEC invasion assays. Number of bacteria associated with Caco-2 and Caco-2/HBD-2 cells was determined by host cell lysis, plating, and counting CFUs/ml. Data are representative of three different experiments ± SD. Significant differences are indicated by ***p<0.001.

**Figure 4:**
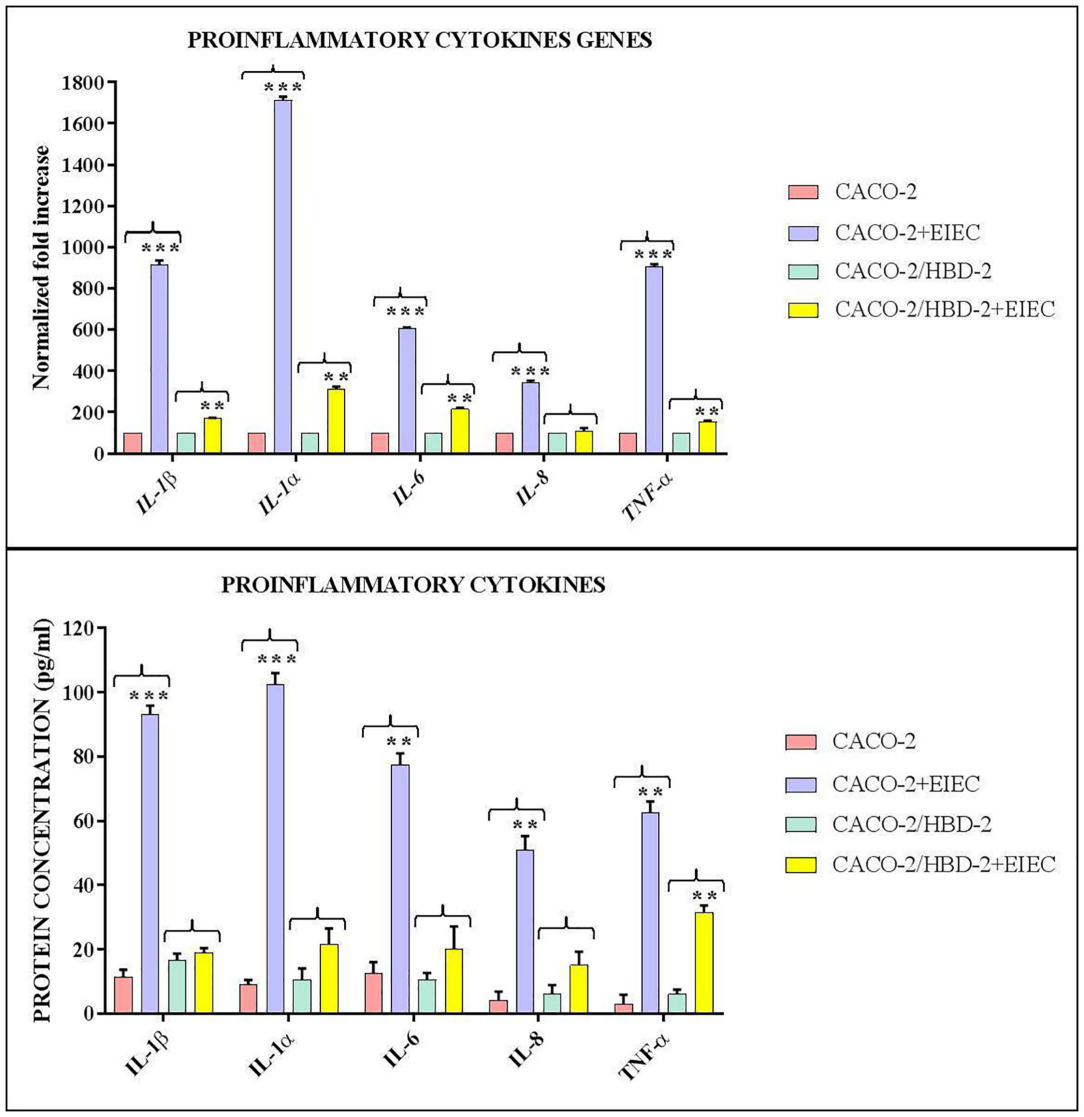
Real-Time PCR results show the effects of HBD-2 on expression of invasion-associated genes in EIEC incubated for 3 hours with Caco-2/HBD-2 and Caco-2 cells, shown as relative gene expression. Data are representative of three different experiments ± SD. Significant differences are indicated by **p<0.01, ***p<0.001.

The CFUs/ml counting carried out following the internalization assay shows (**Figure 3**) that the invasive ability of EIEC decreases by over 99% (1.5 x 10^2^ CFUs/ml) in Caco-2/HBD-2 compared with untransfected Caco-2 (2 x 10^5^ CFUs/ml – *p*<0.001). In addition, the molecular analysis of the expression of bacterial genes associated with invasive capacity, carried out by Real-Time PCR, confirms this trend (**Figure 4**), showing a reduction in the expression of *ipaB* and *ipaC* (*p*< 0.05), but particularly of *ial* and *virB* (respectively *p*<0.01 and< 0.001), in bacteria incubated with Caco-2/HBD-2 compared to those incubated with Caco-2. Only *virF* and *icsA* result unmodulated.

## Discussion

Intestinal homeostasis is regulated by a crosstalk between immune epithelial and mucosal cells and commensal bacteria; the mucosal surface acts as a primary barrier against the invasion of pathogens and the activity of the toxins owing to the presence of tight junctions that physically strengthen the barrier (Fusco et al., 2021) while commensal bacteria create intimate and dynamic interactions with the intestinal epithelium and influence the cellular and immune response of the host by modulating the production of molecules, such as cytokines, chemokines, and AMPs, capable of activating the mechanisms of the innate immune system (Diamond et al., 2009; Lai and Gallo, 2009); however, enteric pathogens have evolved strategies to infiltrate and bypass earlier barriers, among which EIEC, one of the most important *E. coli* pathotypes (Petri et al., 2008). In this work, we evaluated the effect of HBD-2 on the pathogenicity of EIEC in an experimental model of HBD-2- transfected Caco-2 cells.

### HBD-2 activity on EIEC- induced inflammation and on cell viability

It is know that bacterial infections develop through a common four-step infection strategy, whose final result is the induction tissue damage (Boudeau et al., 1999). This induction is due to a locally release of a various inflammatory factors, among which the proinflammatory cytokines.

In the first part of this work, the ability of HBD-2 to reduce the inflammatory state of the intestinal epithelium in EIEC infections was evaluated. The results obtained showed that in HBD-2-transfected cells, the expression of proinflammatory cytokines was significantly lower than in untransfected cells, and the Alamar Blue assay confirmed that their metabolic activity was higher.

### HBD-2 activity on EIEC invasiveness and invasion-related genes expression

The pathogenic strains of *E. coli* act by three general paradigms of virulence: (i) production of enterotoxins, (ii) intimate binding with membrane signaling, and/or (iii) invasion (Cho et al., 2010).

Based on the preliminary data obtained, we have hypothesized that the reduction in the inflammatory state could be related to a decrease in the number of cell-associated bacteria; hence, invasiveness tests were carried out. As expected, the data showed an almost total reduction in the invasive ability of EIEC in transfected cells (> 99%).

It is known that the invasion ability of EIEC is associated with the expression of some virulence genes located on a large plasmid called *inv*, which represents an essential determinant of virulence involved in adhesion, host cell invasion, and cell-to-cell diffusion (Farajzadeh-Sheikh et al., 2020).

In the second part of this work, we examined the role of HBD-2 in the invasive-related gene expression. In particular, we analyzed the expression of the gene encoding the plasmid antigen invasion (*ipa*), involved in bacterial escape from the phagosome and its lysis (Harrington et al., 2006), *virF* and *virB*, which regulate the transcription of *ipa* (Emanuele et al., 2014) and the genes encoding the proteins of type III secretion system (TTSS) (Schroeder and Hilbi, 2008); *icA* and *icsB* are associated with intracellular movement; the former is able to lead bacterial invasion and intracellular spread, and the latter helps escape autophagy (Pasqua et al., 2017). The locus associated with invasion – (*ial*) mediates the response of host to intercellular dissemination (Bando et al., 2010). The results obtained from our experiments show that the presence of HBD-2 in transfected cells can also strongly reduce the expression of the *virB* and *ial* genes, and more weakly, also of the *ipaB* and *ipaC* genes, thus reducing the invasiveness of EIEC and consequently its virulence.

## Conclusions

The progressive onset of multi-drug resistant pathogens poses a big threat to global human health. All antibiotics commonly used in therapy affect bacterial viability, and emerging resistant strains have developed the ability to also survive in the presence of antibiotics with a selective growth advantage. Thus, a potential alternative method to combat bacterial infection is to target virulence factors instead of bacterial viability so as to reduce the frequency of the onset of multidrug-resistant strains. The difference in the expression of the virulence factors is linked to the manifestation of different clinical phenotypes, which can stimulate the host’s immune system in different ways; therefore, understanding their distribution could be useful in designing new antibacterial therapies, targeting specific characters of virulence present only in pathogenic strains, that do not damage the microbiota.

In many Gram-negative bacteria, the expression of virulence factors is regulated by quorum sensing, which depends on the bacterial density. It has been reported that AMPs at sub-lethal concentrations may serve a specific role in modulating *in vitro* the virulence of pathogens (Uhlmann et al., 2016; Vasilchenko and Rogozhin, 2019) as they are able to interfere with this mechanism through a process called “quorum quenching” (Duperthuy, 2020). In this way, they are able to inhibit both the expression of the genes involved in bacterial resistance and virulence and the communication itself between cells, resulting in a specific bacterial response to the stress induced by the presence of AMPs (Emanuele et al., 2014).

In the last years, many studies have focused on analyzing the effective activity of AMPs against multi-drug resistant bacteria, and the results have been decidedly encouraging (Dean et al., 2011; Bolatchiev, 2020; Bolatchiev, 2022), to the point that some AMPs have been used in pre-clinical trials and approved for clinical use (Dijksteel et al., 2021). In this work, we wanted to contribute to enrich this wealth of information by demonstrating that the HBD-2 is active *in vitro* also against EIEC, in fact is able to reduce its pathogenesis through the inhibition of invasiveness and virulence factors’ expression, as already reported for biofilm-associated genes of *S. aureus* and *P. aeruginosa* (Fusco et al., 2021). Hence, we can state that *in vitro* studies on the activity of AMPs can be a valid starting point for clinical investigations on their potential use for innovative therapeutic approaches.

## Data availability statement

The original contributions presented in the study are included in the article/Supplementary Material. Further inquiries can be directed to the corresponding author.

## Author contributions

AF and GD designed the study. AF, VS, and BP oversaw the laboratory procedures. AF wrote the manuscript. GD and RM supervised and validated the original draft. All the authors read and approved the final manuscript.

## Conflict of interest

The authors declare that the research was conducted in the absence of any commercial or financial relationships that could be construed as a potential conflict of interest.

## Publisher’s note

All claims expressed in this article are solely those of the authors and do not necessarily represent those of their affiliated organizations, or those of the publisher, the editors and the reviewers. Any product that may be evaluated in this article, or claim that may be made by its manufacturer, is not guaranteed or endorsed by the publisher.
